# The effects of double gelatin containing chitosan nanoparticles–calcium alginate coatings on the stability of chicken breast meat

**DOI:** 10.1002/fsn3.3686

**Published:** 2023-09-15

**Authors:** Rashid Safari, Milad Yaghoubi, Monika Marcinkowska‐Lesiak, Hamid Paya, Xiaohong Sun, Anahita Rastgoo, Mirmehdi Rafiee, Kazem Alirezalu

**Affiliations:** ^1^ Department of Animal Science, Ahar Faculty of Agriculture and Natural Resources University of Tabriz Tabriz Iran; ^2^ Department of Food Science and Technology, Faculty of Agriculture University of Tabriz Tabriz Iran; ^3^ Department of Technique and Food Development, Institute of Human Nutrition Sciences Warsaw University of Life Sciences Warsaw Poland; ^4^ Department of Animal Science, Faculty of Agriculture University of Tabriz Tabriz Iran; ^5^ Department of Plant, Food, and Environmental Sciences, Faculty of Agriculture Dalhousie University Truro Nova Scotia Canada; ^6^ Department of Food Science and Technology, Faculty of Agriculture Azad University of Khoy Khoy Iran

**Keywords:** antimicrobial coatings, meat, nanoparticle, packaging, shelf life

## Abstract

The effects of gelatin coatings (2% and 4%) containing chitosan nanoparticles (ChNPs; 1% and 2%), in combination with calcium‐alginate coatings (CA; 2%), on quality attributes and shelf life of chicken breast meat were evaluated at 4°C for 12 days. The results indicated that double‐active gelatin–calcium alginate coatings had significant (*p* < .05) effects on moisture and protein content. Incorporation of ChNPs into double gelatin‐CA coatings led to significant reduction (*p* < .05) in TBARS, pH, and TVB‐N values at the end of storage. The counts of total viable count (TVC), coliforms, yeasts, and molds were significantly (*p* < .05) lower in all coated samples, particularly in treated samples by 4% gelatin containing 2% ChNPs + 2% CA coatings (6.85, 6.78, and 5.91 log CFU/g, respectively, compared with 8.35, 8.76, and 7.71 log CFU/g in control) at the end of keeping time. The results of sensory attributes showed that the coated samples had higher overall acceptability scores compared with the untreated samples. A synergistic relationship between the concentrations of gelatin and ChNPs was observed in maintaining the quality characteristics of meat samples during storage. Therefore, this study aims to evaluate the performance of double gelatin coating containing ChNPs in combination with CA coating in the storage quality improvement of chicken breast meat stored for 12 days at 4 °C to develop novel and practical coatings for meat and meat products.

## Graphical abstract

The objective of the present study was to assess the effects of a gelatin coating containing chitosan nanoparticles in combination with a calcium‐alginate coating on the shelf life and quality attributes of chicken breast meat. Incorporation of ChNPs into double gelatin‐CA coatings led to a significant reduction in thiobarbituric acid reactive substances, pH, and total volatile basic nitrogen at the end of storage. All coated samples displayed higher inhibitory effects against microbial growth compared with the control. Based on the obtained results, the incorporation of ChNPs into double gelatin and CA coatings could be utilized as an efficient technique for improving the stability and shelf life of chicken breast meat by day 6.

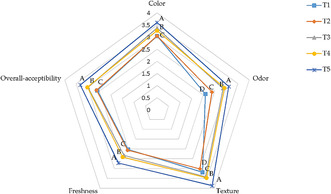

## 1 INTRODUCTION

Chicken meat is known as a rich source of proteins, essential amino acids, and unsaturated fatty acids with a low production cost (Noori et al., 2018). Chicken meat is highly susceptible to oxidation reactions (especially lipid oxidation) and microbial contamination because of its high polyunsaturated fatty acid content, and this is the main reason for its low shelf life (Kerry et al., 2006; Latou et al., 2014). Common pathogenic bacteria that contaminate chicken meat, including *Salmonella* spp., *Campylobacter jejuni*, *Listeria monocytogenes*, and *Escherichia coli* O157: H7, can cause severe foodborne illnesses and even be life‐threatening (Schirone & Visciano, 2021; Woraprayote et al., 2016). Currently, the meat industry utilizes synthetic additives such as sodium nitrite/nitrate to control microbial growth as well as lipid and protein oxidation reactions (Sebranek & Bacus, 2007). However, synthetic additives have health concerns which cannot meet the growing consumer demand for clean label products. Hence, researchers have been widely focused on developing natural additives and preservatives like organic acids, bacteriocins, essential oils, plant extracts, and chitosan by using direct addition or active packaging (Latou et al., 2014; Raeisi et al., 2016; Takma & Korel, 2019; Yaghoubi et al., 2021).

Edible films and coatings, as novel and efficient approaches, particularly in combination with natural preservatives to improve shelf life of meat and meat products, are mainly derived from polysaccharides (e.g., gum, alginate, and chitosan) and proteins (e.g., zein and whey; Dehghani et al., 2018; Javaherzadeh et al., 2020; Liu et al., 2023; Yu et al., 2019). Gelatin is known as one of the most practical coating materials because of its low permeability against oil, aroma, and gas and its good gelling and film‐forming properties (Ramos et al., 2016; Yu et al., 2019). Alginate, inasmuch as it has special colloidal properties like good film‐forming ability, gel production, thickening, and low price, high availability, and biodegradability, is widely utilized as a biodegradable coating and film (Draget et al., 2005). Chitosan, because of its unique film‐forming ability, low gas permeability, good mechanical and biocompatibility properties (Elsabee & Abdou, 2013), nontoxicity properties (Cui et al., 2017), and more importantly, because it possesses strong antioxidant attributes (Alirezalu, Yaghoubi, Nemati, et al., 2021; Alirezalu, Yaghoubi, Poorsharif, et al., 2021) and antimicrobial activities against a wide range of bacteria (Marin‐Silva et al., 2023; Yaghoubi et al., 2021), is widely used in meat and meat products.

Some researchers have reported the antimicrobial properties and antioxidant effects of gelatin‐chitosan coatings in different food products. According to results obtained by Qiu et al. (2022), nanoemulsion‐based chicken bone gelatin–chitosan coatings + natural EOs prolonged the shelf life of chicken patties by more than 4 days. Xiong et al. (2021) also reported that the combination of gelatin + chitosan + gallic acid improved the antioxidant activity of the coating. Gelatin/chitosan coating and hydrolysate have been widely used as natural antimicrobial and antioxidant compounds in meat products like pork meat (Lu et al., 2022), black tiger shrimp (Nagarajan et al., 2021), pork meat (Zhang et al., 2020), minced meat (Pereira et al., 2023), and chicken breast fillet (Hassan et al., 2022).

According to results obtained by some authors, gelatin in combination with chitosan, via hydrogen bonding, can produce a more compact matrix, which can not only effectively improve the softness and flexibility of the coating but also reduce moisture and gas permeability (Pereda et al., 2012). It should be noted that the major limiting factor in the application of gelatin and chitosan as edible coatings is their high moisture permeability due to their inherent hydrophilic nature (Pereda et al., 2012). According to results obtained by Knani et al. (2018)), the increase in bonds between amine groups in gelatin and carboxyls in alginate not only improves the physicochemical parameters of coatings and films (such as thickness and strength) but can also control the permeability of water vapor, oxygen, and carbon dioxide. As a result, double gelatin coating in combination with CA coating could be an efficient technique for maintaining quality attributes and prolonging the shelf life of chicken meat. However, the synergistic effects of gelatin coating containing ChNPs in combination with CA coating were not reported to the best of our knowledge. Therefore, this study aims to evaluate the performance of double gelatin containing chitosan nanoparticles‐calcium alginate coatings in the storage quality improvement of chicken breast meat stored for 12 days at 4 °C to develop a novel and practical coating for meat and meat products.

## 2 MATERIALS AND METHODS

### 2.1 Materials

All microbial mediums and chemical ingredients were provided by Merck. The food grade commercial bovine gelatin powder was purchased from a local store. Sodium alginate and chitosan nanoparticle powder were purchased from Biochemical Company (Shanghai Macklin., Ltd.) and Pishgaman Company, respectively. The chicken breast meats were purchased from a local slaughterhouse (broilers = approximately 3 kg) and transported in ice boxes to the laboratory (the breast meats of broilers separated immediately after slaughter; pH = 5.18). A separate source of boneless and skinless chicken breast meat (3 × 3 × 3 cm) was used in the present work. Individual chicken breasts were weighted and trimmed to approximately 30 g per piece. The samples were evaluated in five batches throughout 3 successive days (five treatments and five time periods with 3 repetitions as well as 3 runs).

### 2.2 Preparation of active antimicrobial coatings

Different concentrations of gelatin (2% and 4%), containing chitosan nanoparticles (ChNPs; 1% and 2%), and stable concentrations of calcium alginate (2%) were prepared according to the methods previously described by Karnjanapratum et al. (2019) and Datta et al. (2008), with some modifications. Two and four grams of gelatin were separately dissolved in sterile distilled water, diluted to 100 mL, and heated in a water bath (at 70°C for 30 min). Throughout the heating, ChNPs at concentrations of 1% and 2% w/w (based on gelatin content) were mixed. Each gelatin + ChNPs solution was separately mixed with Tween 80 (25%) (w/w, based on ChNPs content) as a plasticizer and glycerol (30%) (w/w, based on gelatin content) as an emulsifier. The rotor‐stator homogenizer (Ultra‐Turrax T‐25, IKA Japan Cooperation) was utilized for homogenization (15,000 × *g* for 3 min). Then, calcium alginate coating was made by mixing 0.66 g of calcium carbonate (based on sodium alginate) and 2 g of sodium alginate (Shanghai Macklin Biochemical Co., Ltd.) in distilled water by using mechanical stirring till reaching 100 mL. The solution was autoclaved at 21°C for 20 min and stored at 4°C until used.

### 2.3 Chicken breast meat coating and packaging

The raw materials were randomly divided into five treatments, which are shown in Table 1.

**TABLE 1:** Types and concentrations of the coatings and antimicrobial compounds applied for the treatment of chicken breast meat.

Treatments	Coatings + antimicrobials		
	Calcium alginate (%)	Gelatin (%)	ChNPs (%)
T1 (Control)	–	–	–
T2	2	2	1
T3	2	2	2
T4	2	4	1
T5	2	4	2

Abbreviation: ChNPs, chitosan nanoparticles.

Meat samples were cut into 3 × 3 × 3 cm with a sterile knife and immersed in different concentrations of gelatin (2% and 4%), containing ChNPs (1% and 2%), for 1 h in the refrigerator, drained (5 min), and then immersed in a stable concentration of calcium alginate (2%) for 1 h in the refrigerator. Finally, the meat samples were drained for about 5 min and kept in low‐density polyethylene bags in the refrigerator (4°C) for further analysis at days 1, 3, 6, 9, and 12.

### 2.4 Chemical composition and pH

The protein, fat, moisture, and ash contents of chicken breast meat were evaluated using AOAC (1995) method. Briefly, for moisture content, meat samples were subjected to a gravimetric method using a weight loss experiment in the oven at 105°C until they reached a constant weight. The Soxhlet extraction technique using n‐hexane in an extractor apparatus (liquid–solid extraction) for 1 h (90°C) was used to evaluate fat content. The Kjeldahl total nitrogen method was also utilized for analyzing protein content. The pH of meat samples was measured using a calibrated pH meter (Hanna, Methrom) after mixing 100 mL of distilled water with 10 g of meat samples (Bozkurt & Erkmen, 2007).

### 2.5 Water holding capacity (WHC) and cooking loss

The WHC of chicken breast meat was determined according to the technique described by Ikeda and Foegeding (1999). Five grams of the sample were wrapped in a tissue paper and then centrifuged at 153 × *g* at 25°C for 10 min. WHC was measured by the ratio of water weight after centrifugation to the original content of chicken breast meat water (g) and reported as a percentage of retained water. The cooking loss of meat samples was determined using the standard technique described by Mortensen et al. (2006). Samples were cooked in polyethylene containers at 75°C for 10 min and allowed to cool until they reached 20–25°C. Surface moisture was removed using filter paper, and cooking loss values of the samples were calculated.

### 2.6 Thiobarbituric acid reactive substance (TBARS)

The TBARS values of meat samples were evaluated by using a Spectrophotometer (Hitachi, Ltd.) at 532 nm (Liu et al., 2009). 1,1,3,3‐tetraethoxypropane with 0 to 10 ppm concentrations was used to prepare the standard curve, and the results were expressed as mg MDA/kg of meat samples.

### 2.7 Determination of total volatile nitrogen (TVB‐N)

The Macro Kjeldahl apparatus was utilized for the evaluation of TVB‐N in chicken breast meat samples with vapor distillation (Goulas & Kontominas, 2005). The results were expressed as mg/100 g of meat samples.

### 2.8 Determination of color values

According to Leon et al.'s (2006) method, the lightness (L*), green–red chromaticity (a*), and blue–yellow chromaticity (b*) of meat samples were evaluated using a simple digital imaging system. The meat samples were sized into 10 × 30 × 30 mm thickness for analysis with a calibrated instrument with standard plates. A digital camera was used to capture images under proper lighting at 25°C. The results were evaluated by utilizing MATLAB (The Mathworks, Inc., Version 6.1) software to convert the RGB  L*a*b* values of the meat samples.

### 2.9 Microbiological properties

The microbiological counts of chicken breast meat were performed as follows: Twenty‐five grams of raw samples and 225 mL of peptone water (0.1% w/v; Difco, Becton Dickinson) were mixed by using a sterile lab‐blender (Neutec, Paddle Lab Blender) for 3 min. Peptone water (0.1%) was utilized for serial dilution preparation. Plate Count Agar (PCA) was used for total viable counts (TVC), Violet Red Bile Agar (VRBA) was used for coliform numerations according to the pour plate method, and Dichloran Rose‐Bengal Chloramphenicol Agar (DRBCA) was used for mold and yeast numerations, according to the spread plate method. Coliform and TVC, as well as mold and yeast were kept in incubation for 24 h at 37°C, 48–72 h at 30°C, and 5 days at 25°C, respectively. All data were expressed as log CFU/g of meat (FDA, 2013).

### 2.10 Sensory properties

The impacts of gelatin coating containing ChNPs + CA coating on the sensory properties of chicken breast meat samples were evaluated at day 12. In the present work, 72 consumers with prior experience about fresh meats were adopted as panelists (32 males and 40 females). The organoleptic attributes were performed in six sessions with 12 panelists per sitting. The samples were sized into 3‐mm‐thick cubes, labeled individually with different numbers, and served randomly at room temperature. A hedonic scale (1: really dislike, 5: really like) was employed to evaluate sensory attributes, including color, odor, texture, freshness, and overall acceptability. Between each test, we used water and unsalted crackers to increase the accuracy of the evaluation (Economou et al., 2009).

### 2.11 Statistical analysis

The statistical analysis software (SAS) was used to analyze the obtained data (v .9, SAS Institute). The analyses were carried out in triplicate and averaged for each sample. The variance of data homogeneity and normal distribution has been evaluated in this work. The results of chemical composition were subjected to variance analysis (one‐way ANOVA). Tukey's test was used for the determination of significant differences among means (*p* < .05). Data from the pH, TVB‐N, TBARS, cooking loss, WHC, color values, and microbiological count were analyzed using a RCBD,^1^ considering a mixed linear model with time and treatment as fixed effects and replications as random effects. Results were expressed as mean values ± SE, and statistical significance was revealed with a *p* < .05 value. On day 12, sensory scores (odor, texture, color, freshness, and overall acceptability) were analyzed using a RCBD, and fixed terms for full models included treatments, panelists as a cofactor, and replication (random effects). The least significant difference test determined the potentially significant differences among means (*p* < 0.05; Biffin et al., 2019).

## 3 RESULTS AND DISCUSSION

### 3.1 Chitosan nanoparticle characteristics

Among the quality properties, the size of nanoparticles is the most important parameter, which affects the physical and chemical properties, stability during storage, embedding rate of active substances, bioavailability, as well as technological uses including antimicrobial activities and antioxidant effects. The size of the ChNPs was 50 nm. Azarmi et al. (2008) showed that the successful migration, delivery, and performance of nanoparticles are influenced by their deposition patterns, which are primarily controlled by density and particle size. The purity of ChNPs was higher than 99%, with a density of 1.4 g/cm^3^. Furthermore, the molecular weight of ChNPs in the present work was 161 g/mol.

### 3.2 Chemical composition

The chemical properties of meat samples, including fat and ash, indicated similar values (*p* > .05) except for moisture and protein content (Table 2). The outcomes of this research are not in parallel with those reported by Yaghoubi et al. (2021) on treated chicken meat with a chitosan coating containing *Artemisia fragrance* Eos, which might be due to a difference in coating material type and concentration. Similar results were also expressed by López‐Caballero et al. (2005) on proximate compounds of fish patties coated with a chitosan–gelatin blend.

**TABLE 2:** Chemical composition of chicken breast meat treated with double gelatin containing chitosan nanoparticles + CA coatings.

Chicken breast meat	Properties (%)			
	Moisture	Fat	Ash	Protein
T1	76.39^A^	1.44^A^	1.19^A^	20.64^C^
T2	75.54^AB^	1.37^A^	1.13^A^	21.40^BC^
T3	75.03^BC^	1.39^A^	1.07^A^	21.94^AB^
T4	74.48^C^	1.42^A^	1.09^A^	22.65^A^
T5	74.46^C^	1.36^A^	1.15^A^	22.68^A^
SEM	0.301	0.035	0.026	0.244
*p*‐value	.005	.550	.134	.001

*Note*: T1: control, T2: CA + 2% gelatin + 1% ChNPs, T3: CA + 2% gelatin + 2% ChNPs, T4: CA + 4% gelatin + 1% ChNPs and T5: CA + 4% gelatin + 2% ChNPs. ^A–C^The mean among treatments presented by a different letter is significantly different (*p* < .05).

### 3.3 pH

Microbial growth, which can result in high rates of deterioration, is potentially attributed to pH in meat and meat products (under 6 in fresh meat; Cullere et al., 2018). The results of the samples pH throughout storage are presented in Table 3. The pH in both treated and control samples changed during the keeping period, which might be caused by the formation of lactic acid bacteria (LAB) as well as alkaline compound accumulation (Radha krishnan et al., 2014). However, the formation of nitrogenous components like histamine, tri‐methylamine, ammonia, etc., which are mainly derived from both autolysis by microbial enzymatic actions and endogenous enzymes, is known as one of the other main reasons for increasing pH values during refrigerated storage (Li et al., 2017).

**TABLE 3:** Changes in pH values of chicken breast meat treated with double gelatin containing chitosan nanoparticles + CA coatings during refrigerated storage.

Parameters	Samples	Storage (day)				
		1	3	6	9	12
pH	T1	5.18 ± 0.02^Ae^	5.47 ± 0.02^Ad^	6.11 ± 0.01^Ac^	6.47 ± 0.02^Ab^	6.95 ± 0.02^Aa^
	T2	5.18 ± 0.02^Ad^	5.24 ± 0.02^Bd^	5.71 ± 0.01^Bc^	6.24 ± 0.02^Bb^	6.48 ± 0.02^Ba^
	T3	5.13 ± 0.01^Ad^	5.24 ± 0.01^Bc^	5.44 ± 0.01^Cb^	6.29 ± 0.01^Ba^	6.37 ± 0.01^Ca^
	T4	5.16 ± 0.02^Ae^	5.26 ± 0.01^Bd^	5.44 ± 0.01^Cc^	6.27 ± 0.02^Bb^	6.40 ± 0.01^Ca^
	T5	5.01 ± 0.02^Bd^	5.13 ± 0.01^Cc^	5.15 ± 0.01^Dc^	5.97 ± 0.01^Cb^	6.25 ± 0.01^Da^

*Note*: T1: control, T2: 2% gelatin containing 1% ChNPs + CA, T3: 2% gelatin containing 2% ChNPs + CA, T4: 4% gelatin containing 1% ChNPs + CA and T5: 4% gelatin containing 2% ChNPs + CA. ^a–e^The mean during storage presented by a different letter is significantly different (*p* < .05). ^A–D^The mean between treatments presented by a different letter is significantly different (*p* < .05).

According to Table 3, the rate of pH increase in untreated samples was significantly (*p* < .05) higher than in coated samples with gelatin containing ChNPs + CA. At the end of storage time, coated fillets with T5 coating and uncoated samples displayed the lowest (6.25) and highest (6.95) pH, respectively. Clearly, the control meat samples suffered severe meat deterioration. Low pH levels in treated samples might be related to the potential antimicrobial activity of chitosan. The results of pH are also in agreement with TVB‐N values (Table 3 and Figure 2) among treatments. Similar results are also reported by Liu et al. (2019). The authors evaluated the chilled meat treated with chitosan films and reported a similar trend in pH. Lin et al. (2023) also evaluated the effects of gelatin‐active packaging films loaded with other natural preservatives and reported that, compared with the control group, the pH value of chicken packaged with films changed less. There are other studies that have expressed similar results in chicken meat treated with chitosan combined with natural preservatives (Berizi et al., 2018; Vaithiyanathan et al., 2011). Xiong et al. (2021) showed that the combination of gelatin and chitosan coating incorporated with clove oil had no additional effect on the pH values in fresh salmon fillet, but the incorporation of a high concentration of gallic acid suppressed the increase in the pH, and the combined gelatin and chitosan coating incorporated with clove oil and gallic acid was the most effective in suppressing the increase in the pH values among the samples.

### 3.4 Water holding capacity (WHC) and cooking loss

The economic and sensory properties of meat are attributed to WHC values. Meat oxidation can affect the water holding capacity of myofibrils, leakage, and lightness of meat (Alirezalu, Shafaghi Molan, Yaghoubi, et al., 2021). Therefore, further reduction in oxidation by adding the natural antioxidant can improve WHC, which inhibits leakage and lightness change in the meat. During storage, the WHC of all chicken breast meat samples was remarkably decreased (Table 4). The samples coated with T5 had significantly (*p* < .05) higher WHC values. Gharibzahedi and Mohammadnabi (2017) investigated the effects of jujube gum coating containing nettle oil‐loaded nanoemulsions on the quality properties of Beluga sturgeon fillets and reported improvements in WHC and water barrier characteristics.

**TABLE 4:** Changes in WHC and cooking loss of chicken breast meat treated with double gelatin containing chitosan nanoparticles + CA coatings during refrigerated storage.

Parameters	Samples	Storage (day)			
		1	4	8	12
WHC	T1	59.23 ± 0.52^Ba^	55.33 ± 0.41^Cab^	54.00 ± 1.06^Ab^	51.33 ± 0.75^Bb^
	T2	60.51 ± 0.57^Ba^	59.13 ± 0.96^BCa^	54.27 ± 0.61^Ab^	52.07 ± 0.63^ABb^
	T3	63.43 ± 1.02^ABa^	61.03 ± 1.07^ABa^	56.11 ± 0.30^Ab^	53.17 ± 0.75^ABb^
	T4	66.31 ± 0.44^Aa^	64.60 ± 0.36^Aa^	55.80 ± 0.96^Ab^	55.37 ± 1.33^ABb^
	T5	66.17 ± 0.66^Aa^	64.50 ± 0.35^Aa^	57.70 ± 0.72^Ab^	56.17 ± 0.96^Ab^
Cooking loss	T1	22.81 ± 0.27^Ac^	28.57 ± 0.41^Ab^	32.45 ± 0.40^Aa^	35.57 ± 0.70^Aa^
	T2	21.09 ± 0.54^Ab^	23.52 ± 0.28^Bb^	30.66 ± 0.54^Aa^	32.94 ± 0.29^Aa^
	T3	21.66 ± 0.32^Ab^	22.63 ± 0.53^Bb^	30.27 ± 0.78^Aa^	30.06 ± 0.43^Ba^
	T4	17.48 ± 0.65^Ba^	18.43 ± 0.75^Ca^	26.55 ± 0.83^Bb^	28.70 ± 0.85^BCb^
	T5	17.33 ± 0.63^Bb^	18.12 ± 0.95^Cb^	19.58 ± 0.73^Cb^	25.73 ± 0.58^Ca^

*Note*: T1: control, T2: 2% gelatin containing 1% ChNPs + CA, T3: 2% gelatin containing 2% ChNPs + CA, T4: 4% gelatin containing 1% ChNPs + CA and T5: 4% gelatin containing 2% ChNPs + CA. ^a–c^The mean during storage presented by a different letter is significantly different (*p* < .05). ^A–D^The mean among treatments presented by a different letter is significantly different (*p* < .05).

The cooking loss of the meat samples attributed to its ability to retain water in the food system when protein aggregates and denatures under thermal processing. The cooking loss of all meat samples was increased during the keeping time (Table 4). According to Table 4, samples coated with T5 had the lowest amount of cooking loss compared with other treatments and controls. Meanwhile, although a significant relationship between WHC results and cooking loss, pH, redness, and lightness was reported for beef by Cheng et al. (2019), there was no appropriate relationship between WHC and redness results, which may be due to their negligible changes after coating treatment. In summary, a significant relationship among the WHC pH, and microbial counts occurred to understand the stability changes of the samples during keeping time.

### 3.5 Changes in TBARS value

**FIGURE 1:**
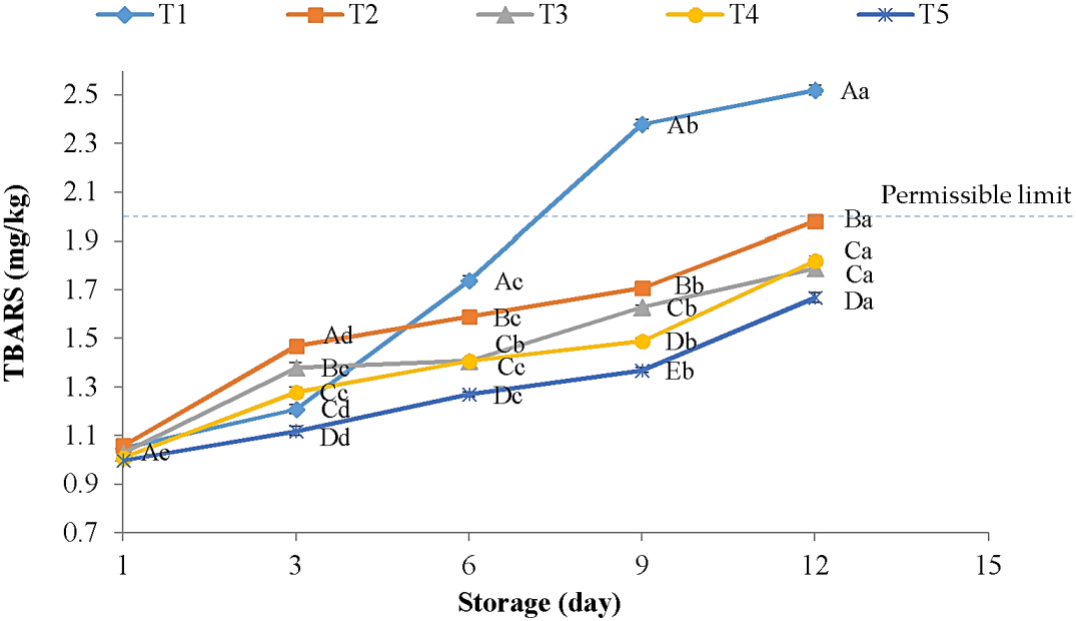
Changes in TBARS (mg/kg) of chicken breast meat treated with double gelatin containing chitosan nanoparticles + CA coatings during refrigerated storage T1: control, T2: 2% gelatin containing 1% ChNPs + CA, T3: 2% gelatin containing 2% ChNPs + CA, T4: 4% gelatin containing 1% ChNPs + CA and T5: 4% gelatin containing 2% ChNPs + CA. ^A–E^Mean values among treatments not followed by a common letter differ significantly (*p* < .05). ^a–e^Mean values not followed by a common letter differ significantly during storage (*p* < .05).

Oxidation reactions, especially in lipids, can potentially affect the stability and quality attributes of meat and meat products, effectively in chicken and fish meat samples (Alirezalu, Hesari, Yaghoubi, et al., 2021; Benjakul et al., 2005). TBARS is known as one of the most pivotal factors in meat and meat products because it shows the production of secondary products (especially aldehydes) by lipid oxidation (Cai et al., 2014; Sun et al., 2019). The effects of gelatin coating containing ChNPs + CA coating on TBARS of chicken breast meat samples are presented in Figure 1.

At day 1, uncoated samples displayed 1.05 mg of MDA/kg of meat, which increased significantly throughout the 12 days of refrigerated period and reached 2.20 mg of MDA/kg of meat. The results also indicated that coated samples with T5 had the lowest TBARS. This might be related to the chitosan coating's oxygen/gas barrier function, which directly limited the exposure of meat to oxygen (Xiong et al., 2020). It was observed that the TBARS of all the double‐active gelatin + CA coated meat samples was much lower on day 12 than the control on day 9 (Figure 1). These results implicitly proved that double‐active coatings delayed the oxidation of lipids for at least 3 days.

Liu et al. (2019) and Jonaidi Jafari et al. (2018) assessed the impacts of chitosan coatings + other natural ingredients and reported similar TBARS results in chilled meat and chicken fillets, respectively. It has been reported that the chitosan chelating effect might be the main reason for low TBARS in treated samples (Sogut & Seydim, 2019). Lin et al. (2023) evaluated the effects of gelatin‐active packaging film loaded with other natural additives and reported similar TBARS results in chicken meat. Fang et al. (2018) and Pabast et al. (2018) also reported similar trends for TBARS levels during keeping time in fresh pork and lamb meat, respectively, treated by chitosan‐based coatings. The high antioxidant attributes of gelatin films containing chitosan were also reported by Sul et al. (2023). As expected, double gelatin coatings containing ChNPs and CA coatings prolonged the shelf life of samples through antioxidative activities. At the end of storage, TBARS values in all samples were within the permissible limit (2 mg MDA/kg) except for control, in which there is no rancidity in chicken breast meat samples (Zhang et al., 2019), revealing that the control meat had the highest degree of lipid oxidation. Therefore, the control samples underwent lipid oxidation due to exposure to oxygen, and the production of free radicals in the lipid chain reaction resulted in further acceleration of oxidation (Xiong et al., 2020). There was a significant relationship between TBARS and the sensory properties of coated chicken breast meat, which may be due to antimicrobial activity and antioxidant effects of ChNPs and the ability to lower the permeability of oxygen/gas in double gelatin + CA coatings (Taşkaya & Yaşar, 2017).

### 3.6 Changes in TVB‐N value

**FIGURE 2:**
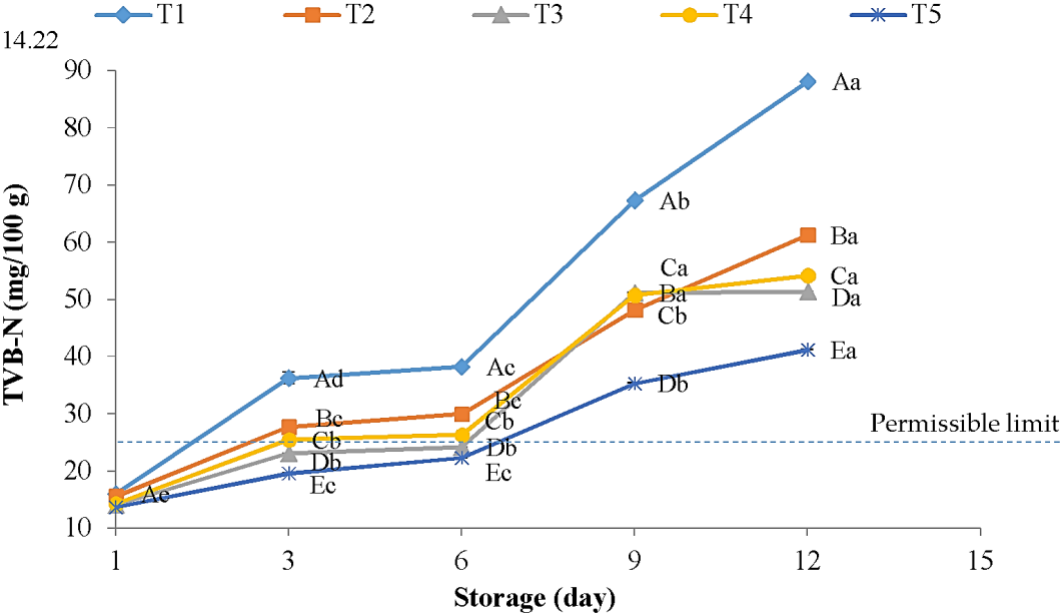
Changes in TVB‐N (mg/100 g) of chicken breast meat treated with double gelatin containing chitosan nanoparticles + CA coatings during refrigerated storage. T1: control, T2: 2% gelatin containing 1% ChNPs + CA, T3: 2% gelatin containing 2% ChNPs + CA, T4: 4% gelatin containing 1% ChNPs + CA and T5: 4% gelatin containing 2% ChNPs + CA. ^A–E^Mean values among treatments not followed by a common letter differ significantly (*p* < .05). ^a–e^Mean values not followed by a common letter differ significantly during storage (*p* < .05).

TVB‐N values are known as one of the most pivotal indicators in evaluating the quality attributes and shelf life of meat and meat products (Ala & Shahbazi, 2019; Alirezalu, Pirouzi, Yaghoubi, et al., 2021). It is mainly produced by enzymatic degradation or spoilage bacteria throughout the keeping time and mainly contains DMA, TMA, and ammonia in the spoilage of meat (Cai et al., 2014). The results of TVB‐N values in chicken breast meats throughout the keeping period are illustrated in Figure 2. At day 1, the TVB‐N values in all meat samples ranged between 13.7 and 16.07 mg/100 g. The TVB‐N values in all treated and untreated samples increased significantly (*p* < .05) during the refrigerated time, and the rate of that was remarkably lower in coated samples compared with control. With regard to meat quality, TVB‐N limit values of 25 mg/100 g of sample for chicken have been proposed as indicators of meat quality (Alirezalu, Pirouzi, Yaghoubi, et al., 2021), indicating microbiological contamination, particularly psychrotrophic bacteria growth, in meat and meat products. It has been shown that the growth of *Enterobacteriaceae* and *Pseudomonas* spp. could explain the changes in TVB‐N values of meat and meat products throughout the keeping period (Balamatsia et al., 2007). The relationship between TVB‐N values and the populations of spoilage bacteria was observed by many authors (Alirezalu et al., 2022; Yaghoubi et al., 2021).

According to the results in Figure 2, it was seen that T3, T4, and T5 remained within acceptable limits on day 3, whereas coated samples with T5 reduce the formation of volatile nitrogenous compounds under acceptable limits until day 6. The TVB‐N values of 22.26 mg/100 g were obtained for T5 coatings, compared with 38.31 mg/100 g for control on day 6. The microbiological results of this study are also in agreement with TVB‐N values. Abdou et al. (2018) and Mojaddar Langroodi et al. (2018) also reported that chitosan, when combined with other natural additives, can reduce TVB‐N production. The antioxidant and antimicrobial properties of the new formulated coating incorporated with ChNPs might be the main reason for the low TVB‐N values in coated samples.

### 3.7 Determination of color values

The color values (L* (Lightness), a* (redness), and b* (yellowness)) of samples were considerably affected by both new coating materials and storage (Table 5). At day 1, all treated meat samples had significantly (*p* < .05) higher L* than the control. All chicken breast meat samples displayed a significant (*p* < .05) decrease in lightness at day 12, and control and treated samples with T3 showed the lowest and highest L* values (27.98 and 34.24, respectively). Oxidative reactions, especially those that occur on lipids, lead to metmyoglobin formation (tan or brown in color), which could decrease L* values during refrigerated storage. As a result, antioxidant compounds decrease oxidation reactions, preventing L* values from rapidly decreasing (Nemati et al., 2021; Yaghoubi et al., 2021). High antioxidant attributes of ChNPs might be the main reason for higher L* values in treated samples compared with controls. A similar result was observed by Yaghoubi et al. (2021) on chicken meat samples coated with chitosan containing *Artemisia fragrans* essential oil and Alirezalu et al. (2022) on chicken meat samples coated with calcium alginate containing *Artemisia fragrans* essential oil. There are many factors, including microbial growth, protein and lipid oxidation, and pH, that can affect the L* values of meat and meat products (Yu et al., 2019). The color attributes of coated chicken meat samples indicated that the reduction in L* values can be effectively prevented, and this might be related to the direct antioxidant and antibacterial protection from the coating (Wang et al., 2018; Yu et al., 2019). Chitosan and gelatin in combination can produce a more compact coating structure (Pereda et al., 2012), and additional protective effects can be obtained by double coating, both of which may further improve the preservative impact of the coating (Fang et al., 2018). In red meat like pork and beef, the a* value (redness) is a common factor to evaluate the freshness, and a higher a* value generally shows meat freshness, but in chicken meat, this parameter varies greatly among the researches and is potentially dependent on species (Mancini & Hunt, 2005; Yu et al., 2019). All coatings preserved the chicken meat from discoloration. Coated samples with T3 showed the highest a* value compared with control, which might be related to the high antioxidant properties of the new coating, suggesting slight oxidation and discoloration. The oxidation of myoglobin to met‐myoglobin as a result of direct exposure of meat to oxygen in control samples may be the reason for the darker color change of untreated chicken meat samples throughout the keeping time in comparison with treated samples. Therefore, the double coating acted as a barrier to oxygen and resulted in reduced myoglobin oxidation. The T3 coatings displayed the highest protection among all coatings, which might be related to the joint antibacterial and antioxidant activity of the ChNPs and the controlled moisture and gas barrier of the composite coating (Fang et al., 2018). The similar results observed by De Carvalho et al. (2019) in treated lamb burgers with natural plant extracts. The production of met‐myoglobin (as a result of protein and lipid oxidation reactions) might be the main reason for a* reduction throughout the refrigerated period (Ayaseh et al., 2022; Zhang et al., 2020).

**TABLE 5:** Changes in color indexes of chicken breast meat treated with double gelatin containing chitosan nanoparticles + CA coatings during refrigerated storage.

Treatments		Storage (day)				
		1	3	6	9	12
L*	T1	30.82 ± 0.18^Ca^	28.90 ± 0.58^Bab^	26.94 ± 0.53^Bb^	27.78 ± 0.31^Cb^	27.98 ± 0.31^Cab^
	T2	31.16 ± 0.44^Ca^	25.75 ± 1.38^Bb^	33.0 ± 0.01^Aa^	33.81 ± 0.03^Ba^	29.16 ± 0.26^BCa^
	T3	33.44 ± 0.26^Bb^	37.20 ± 0.18^Aa^	34.33 ± 0.34^Ab^	36.68 ± 0.93^Aa^	34.24 ± 0.65^Ab^
	T4	36.83 ± 0.35^Aa^	37.59 ± 0.28^Aa^	35.50 ± 0.01^Aa^	36.88 ± 0.50^Aa^	32.67 ± 0.55^ABb^
	T5	37.15 ± 0.19^Aa^	36.56 ± 0.64^Aa^	34.44 ± 0.43^Aab^	30.82 ± 0.44^BCb^	31.90 ± 0.36^Bb^
a*	T1	0.58 ± 0.18^Ab^	5.51 ± 0.38^Aa^	−0.85 ± 0.14^Abc^	−2.60 ± 0.71^Ac^	−7.50 ± 0.36^Cd^
	T2	−1.35 ± 0.05^Bb^	−2.95 ± 0.25^Ba^	−3.73 ± 0.18^Bc^	−3.02 ± 0.17^Ad^	−4.19 ± 0.12^Bc^
	T3	−3.59 ± 0.12^Cab^	−3.75 ± 0.04^Bab^	−4.74 ± 0.31^Bb^	−3.52 ± 0.23^Aa^	−2.37 ± 0.31^Aa^
	T4	−1.81 ± 0.03^Ba^	−3.87 ± 0.28^Bb^	−7.45 ± 0.27^Cd^	−2.31 ± 0.04^Aa^	−5.35 ± 0.57^Bc^
	T5	−3.71 ± 0.08^Cbc^	−2.02 ± 0.20^Bab^	−0.80 ± 0.11^Aa^	−2.36 ± 0.33^Ab^	−4.75 ± 0.50^Bc^
b*	T1	19.07 ± 1.10^BCab^	19.26 ± 0.26^Ca^	17.91 ± 0.25^BCb^	16.32 ± 0.35^Bb^	20.59 ± 0.23^Ca^
	T2	18.67 ± 0.16^Ca^	20.39 ± 0.37^BCab^	21.26 ± 0.03^Ac^	21.65 ± 0.30^Ab^	22.85 ± 0.50^BCc^
	T3	17.02 ± 0.42^Cc^	23.46 ± 0.30^Aab^	21.41 ± 0.68^Ab^	21.56 ± 0.18^Ab^	23.20 ± 0.33^ABa^
	T4	20.81 ± 0.53^ABa^	21.37 ± 0.08^Ba^	22.11 ± 0.46^Aa^	20.30 ± 0.11^Aa^	22.68 ± 0.10^BCa^
	T5	22.20 ± 0.51^Aa^	20.24 ± 0.31^BCb^	20.56 ± 0.10^ABb^	19.93 ± 0.47^Ab^	23.80 ± 0.30^ABa^

*Note*: T1: control, T2: 2% gelatin containing 1% ChNPs + CA, T3: 2% gelatin containing 2% ChNPs + CA, T4: 4% gelatin containing 1% ChNPs + CA and T5: 4% gelatin containing 2% ChNPs + CA. ^a–d^The mean during storage presented by a different letter is significantly different (*p* < .05). ^A–D^The mean among treatments presented by a different letter is significantly different (*p* < .05).

The b* values (yellowness) of all treated and untreated meat samples gradually increased over the storage period, which might be related to the enzymatic browning reaction of phenolic components. The higher yellowness is often related to the accumulation of methemoglobin and metmyoglobin and high levels of lipid oxidation (Xiong et al., 2020). Chicken meat tends to become yellower during storage period as it deteriorates (Wang et al., 2018). In the present work, the coatings indicated a remarkable impact on the yellowness until day 12, and all the treated meat samples had higher b* values than the control (*p* < .05). The results suggested that all coatings altered yellowness of the chicken breast meat efficiently, although no obvious trend was found among the coated meat samples. The b* values of meat samples were also influenced by the enzymatic browning reaction of phenolic components. The control sample displayed the lowest b* values.

### 3.8 Microbiological properties

The microbial count (TVC, coliforms, molds, and yeasts) of samples revealed potential antimicrobial properties of gelatin containing ChNPs + CA coatings (Table 6). On day 1, the TVC in coated samples (3.06–3.57 log CFU/g) was significantly (*p* < .05) lower than in uncoated samples (4.52 log CFU/g).

**TABLE 6:** Changes in microbiological counts (log CFU/g) of chicken breast meat treated with double gelatin containing chitosan nanoparticles + CA coatings during refrigerated storage.

Micro‐organism	Treatments	Storage (day)				
		1	3	6	9	12
Total viable count	T1	4.52 ± 0.03^Ae^	5.92 ± 0.03^Ad^	7.83 ± 0.03^Ac^	8.02 ± 0.03^Ab^	8.35 ± 0.02^Aa^
	T2	3.57 ± 0.02^Be^	5.01 ± 0.03^Bd^	5.64 ± 0.03^Bc^	7.11 ± 0.02^Bb^	7.51 ± 0.03^Ba^
	T3	3.27 ± 0.03^Ce^	4.85 ± 0.03^Cd^	5.35 ± 0.02^Cc^	6.23 ± 0.03^Cb^	7.12 ± 0.03^Ca^
	T4	3.41 ± 0.03^Be^	4.76 ± 0.02^Cd^	5.27 ± 0.02^Cc^	6.01 ± 0.03^Db^	7.25 ± 0.02^Ca^
	T5	3.06 ± 0.03^Dd^	4.31 ± 0.02^Dd^	5.01 ± 0.03^Dc^	5.97 ± 0.02^Db^	6.85 ± 0.03^Da^
Coliforms	T1	4.25 ± 0.03^Ae^	4.95 ± 0.02^Ad^	7.52 ± 0.03^Ac^	8.23 ± 0.03^Ab^	8.76 ± 0.03^Aa^
	T2	4.18 ± 0.03^Ae^	4.72 ± 0.03^Bd^	6.81 ± 0.02^Bc^	7.30 ± 0.03^Bb^	7.67 ± 0.02^Ba^
	T3	3.21 ± 0.03^Ce^	4.18 ± 0.02^Dd^	5.98 ± 0.03^Dc^	6.74 ± 0.03^Cb^	7.18 ± 0.02^Ca^
	T4	3.77 ± 0.02^Be^	4.34 ± 0.03^Cd^	6.45 ± 0.02^Cc^	6.94 ± 0.03^Cb^	7.28 ± 0.02^Ca^
	T5	2.94 ± 0.03^De^	3.98 ± 0.03^Dd^	5.49 ± 0.03^Ec^	6.18 ± 0.02^Db^	6.78 ± 0.03^Da^
Molds and yeasts	T1	3.44 ± 0.02^Ae^	3.75 ± 0.02^Ad^	6.18 ± 0.02^Ac^	7.41 ± 0.02^Ab^	7.71 ± 0.02^Aa^
	T2	2.94 ± 0.01^Be^	3.21 ± 0.02^Bd^	4.95 ± 0.02^Bc^	5.67 ± 0.02^Bb^	6.61 ± 0.02^Ba^
	T3	2.34 ± 0.02^Ce^	2.88 ± 0.02^Cd^	4.45 ± 0.02^Cc^	5.38 ± 0.02^Cb^	6.27 ± 0.02^Ca^
	T4	2.44 ± 0.02^Ce^	2.68 ± 0.02^Dd^	4.51 ± 0.02^Cc^	5.17 ± 0.02^Db^	6.35 ± 0.01^Ca^
	T5	2.01 ± 0.02^De^	2.31 ± 0.02^Ed^	4.18 ± 0.02^Dc^	4.87 ± 0.02^Eb^	5.91 ± 0.02^Da^

*Note*: T1: control, T2: 2% gelatin containing 1% ChNPs + CA, T3: 2% gelatin containing 2% ChNPs + CA, T4: 4% gelatin containing 1% ChNPs + CA and T5: 4% gelatin containing 2% ChNPs + CA. ^a–e^The mean during storage presented by a different letter is significantly different (*p* < .05). ^A–E^The mean among treatments presented by a different letter is significantly different (*p* < .05).

In fresh meat, 7 log CFU/g is the acceptable limitation of TVC (Yu et al., 2019). The TVC of all chicken samples increased remarkably throughout the keeping period but remained under the permissible limit for 9 days of refrigerated storage, except for control and T2. On day 12, all treated and untreated samples exceeded this limit except chicken meat coated with T5. The uncoated samples showed the fastest increase (7.83 log CFU/g) in TVC on day 6, meaning that the control meat had high microbial spoilage. The gelatin–calcium alginate‐coated meat sample also revealed a fast increase in TVC, but to a lesser extent than the control sample, with 7.11 log CFU/g on day 9. Although gelatin in combination with calcium alginate has no antimicrobial properties, a physical barrier against microorganisms may be provided by coating (Ramos et al., 2016). According to results obtained by Min and Oh (2009), gelatin coating alone in catfish flesh remarkably inhibited the growth of G‐negative bacteria, like *Escherichia coli* O157:H7 and *Salmonella typhimurium*, during refrigerated time. Unsimilar to the poor impact of gelatin on TVC, chitosan nanoparticles incorporated in gelatin + CA coatings maintained TVC under the acceptable limit (<7 log/g) among 6 days of refrigerated storage, and their TVC on day 12 was lower or close to that of the control meat on day 6. These results suggested that gelatin coatings containing ChNPs in combination with calcium alginate coatings could potentially delay the microbial spoilage of the chicken meat samples by about 6 days. Chitosan as a strong antimicrobial ingredient has been demonstrated to inhibit the growth of a range spectrum of G‐negative bacteria (like *Pseudomonas* spp., *Vibrio* spp., and *Escherichia coli*), G‐positive bacteria (*Lactobacillus* spp., *Listeria monocytogenes*, and *Staphylococcus aureus*), and fungi (like *Botrytis cinerea* and *Drechstera sorokiana*; Goy et al., 2009). The high antimicrobial activities of chitosan in coated meat products were also reported by Elsabee and Abdou (2013). Among the chitosan‐based coatings, no remarkable difference (*p* > .05), or any trend, was obtained among samples coated with T3 and T4 during the keeping time. These results indicated that the combination of gelatin and calcium alginate or the incorporation of ChNPs may have a further antimicrobial impact on the double coating. The TVC value of treated samples with gelatin‐CA coatings containing high concentrations of ChNPs was significantly (*p* < .05) lower than the control, and samples coated with 4% gelatin‐CA being the lowest among all meat samples during the storage period. The outcomes of the present work are in agreement with Bazargani‐Gilani et al. (2015). The authors assessed the impacts of chitosan in combination with *Zataria multiflora Boiss* essential oil and reported a 10‐day storage time extension in chicken breast meat.

As hygienic quality indicator, coliforms are listed among the most important microorganisms in meat and meat products (Kunová et al., 2014). Gelatin coatings containing ChNPs in combination with CA coatings displayed potential inhibitory effects against coliforms. Coliform bacteria increased significantly (*p* < .05) during 12 days of cold storage. However, the rate of coliforms in control samples increased faster than in treated samples throughout keeping period. Mold and yeasts, which grow highly on meat and meat products surfaces, can potentially result in spoilage and negative effects on quality attributes. During the 12‐day refrigerated period, coated samples showed higher inhibitory effects than uncoated samples against mold and yeast, particularly in samples coated with T5. The initial count of mold and yeasts ranged between 2.01 and 3.44 log CFU/g for treated meat samples with T5 and control samples, respectively, which reached 5.91 and 7.71 log CFU/g at day 12. The outcomes of this research revealed that T5 coating had the highest antimicrobial properties against coliforms, mold, and yeasts, as well as TVC, in chicken meat samples. The results of this work are in agreement with Yaghoubi et al. (2021) and Berizi et al. (2018). These authors also reported similar antimicrobial properties in treated chicken fillets and rainbow trout coated with chitosan in combination with *Artemisia fragrance* essential oils and pomegranate peel extract, respectively. Abdel Aziz and Salama (2021) evaluated edible coatings based on calcium alginate in combination with aloe vera and garlic oil and reported significant antimicrobial properties of CA coatings against G‐negative and G‐positive bacteria. Liu et al. (2023) also assessed the effects of a self‐assembled carboxymethyl chitosan/zinc alginate composite film on the quality attributes of pork meat. The authors reported high antimicrobial attributes of alginate films in the presence of chitosan/zinc against spoilage bacteria. The high antimicrobial attributes of chitosan nanoparticles in beef meat were also reported by Lotfy et al. (2023).

### 3.9 Sensory properties

**FIGURE 3:**
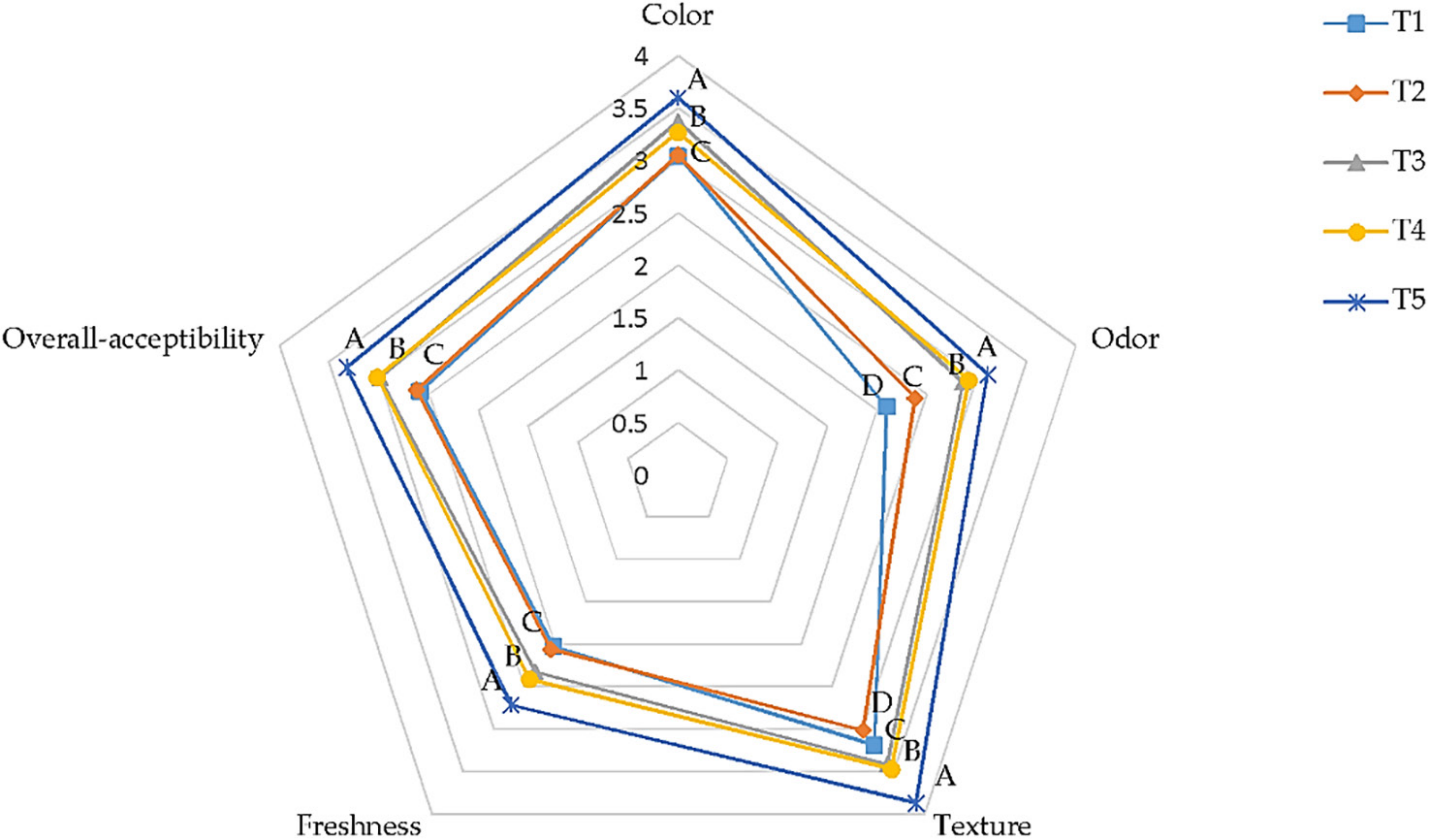
Sensory attributes of chicken breast meat treated with double gelatin containing chitosan nanoparticles + CA coatings on day 12. T1: control, T2: 2% gelatin containing 1% ChNPs + CA, T3: 2% gelatin containing 2% ChNPs + CA, T4: 4% gelatin containing 1% ChNPs + CA and T5: 4% gelatin containing 2% ChNPs + CA. ^A–D^The mean among treatments presented by a different letter is significantly different (*p* < .05).

Sensory attributes of all chicken breast meat samples (including color, odor, texture, freshness, and overall acceptability) were assessed using a 5‐point hedonic scale at the end of the storage period. The effects of gelatin coating containing ChNPs in combination with CA coating are presented in Figure 3. The results of the present research indicated that coated samples with T5 displayed the highest scores (*p* < .05), while the lowest scores were observed in controls. The results indicated that new formulated coatings had significantly (*p* < .05) positive effects on chicken meat colors. The odor, texture, freshness, and overall acceptability were lower in uncoated samples. The low scores for uncoated samples may be related to the high level of oxidation reactions and microbial growth at the end of storage.

Yaghoubi et al. (2021) reported similar sensory effects in chicken meat coated with chitosan coating + *Artemisia fragrance* essential oils. The results of this research are also in agreement with ready‐to‐cook poultry products (Giatrakou et al., 2010) coated with thyme and chicken breast meat coated with chitosan containing oregano oil, either singly or combined in a modified atmosphere package (Petrou et al., 2012).

## 4 CONCLUSION

The results for microbiological count and chemical composition revealed that gelatin coatings containing ChNPs in combination with CA coating on chicken breast meat can lead to good quality properties, acceptable sensory attributes, enhancement of microbiological safety, and improvement of shelf life during storage periods. All treatments declined remarkably in microbial counts when compared with the control. The quality attributes (TVB‐N, TBARS, and pH) of coated samples remained within acceptable range for a longer period, approximately on day 6. The highest antimicrobial and antioxidant properties were observed in samples coated with 4% gelatin containing 2% ChNPs + CA. The outcomes of the present work revealed that the shelf life of chicken breast meat could be significantly increased by double‐active gelatin + calcium alginate coatings in combination with ChNPs, which can be suggested as potential coating materials.

## AUTHOR CONTRIBUTIONS

**Rashid Safari:** Conceptualization (equal); writing – review and editing (equal). **Milad Yaghoubi:** Formal analysis (lead); writing – original draft (lead). **Monika Marcinkowska‐Lesiak:** Writing – review and editing (equal). **Hamid Paya:** Formal analysis (equal); writing – original draft (equal). **Xiaohong Sun:** Writing – review and editing (equal). **Anahita Rastgoo:** Formal analysis (equal). **Mirmehdi Rafiee:** Formal analysis (equal). **Kazem Alirezalu:** Conceptualization (lead); writing – original draft (equal); writing – review and editing (equal).

## CONFLICT OF INTEREST STATEMENT

The authors declare no conflict of interest relevant to this article.

## ETHICS STATEMENT

This article does not cover any human or animal studies conducted by any of the authors. Not applicable.

## Data Availability

The data are available upon request from the authors.

## Footnotes

^1^
Randomized complete block design.
